# Clinical utility of periodic reinterpretation of CNVs of uncertain significance: an 8-year retrospective study

**DOI:** 10.1186/s13073-023-01191-6

**Published:** 2023-05-23

**Authors:** Jean-Marie Ravel, Mathilde Renaud, Jean Muller, Aurélie Becker, Émeline Renard, Thomas Remen, Geneviève Lefort, Mylène Dexheimer, Philippe Jonveaux, Bruno Leheup, Céline Bonnet, Laëtitia Lambert

**Affiliations:** 1 Service de génétique médicale, CHRU de Nancy, Nancy, France; 2 Laboratoire de génétique médicale, CHRU Nancy, Nancy, France; 3 Université de Lorraine, NGERE F-54000Nancy Inserm, France; 4 Laboratoires de Diagnostic Génétique, Institut de Génétique Médicale d’Alsace (IGMA), Hôpitaux Universitaires de Strasbourg, Strasbourg, France; 5 Laboratoire de Génétique Médicale, INSERM, UMRS_1112, Institut de Génétique Médicale d’Alsace (IGMA), Université de Strasbourg Faculté de Médecine de Strasbourg, 67000 Strasbourg, France; 6 Unité Fonctionnelle de Bioinformatique Médicale Appliquée au Diagnostic (UF7363), Hôpitaux Universitaires de Strasbourg, 67000 Strasbourg, France; 7 Department of pediatrics, Regional University Hospital of Nancy, Allée du Morvan, 54511 Vandoeuvre-Lès-Nancy, France; 8 UMDS, CHRU Nancy, Nancy, France

**Keywords:** Array-CGH, Copy-number variation, CNV, VUS reinterpretation, ACMG criteria

## Abstract

**Background:**

Array-CGH is the first-tier genetic test both in pre- and postnatal developmental disorders worldwide. Variants of uncertain significance (VUS) represent around 10~15% of reported copy number variants (CNVs). Even though VUS reanalysis has become usual in practice, no long-term study regarding CNV reinterpretation has been reported.

**Methods:**

This retrospective study examined 1641 CGH arrays performed over 8 years (2010–2017) to demonstrate the contribution of periodically re-analyzing CNVs of uncertain significance. CNVs were classified using AnnotSV on the one hand and manually curated on the other hand. The classification was based on the 2020 American College of Medical Genetics (ACMG) criteria.

**Results:**

Of the 1641 array-CGH analyzed, 259 (15.7%) showed at least one CNV initially reported as of uncertain significance. After reinterpretation, 106 of the 259 patients (40.9%) changed categories, and 12 of 259 (4.6%) had a VUS reclassified to likely pathogenic or pathogenic. Six were predisposing factors for neurodevelopmental disorder/autism spectrum disorder (ASD). CNV type (gain or loss) does not seem to impact the reclassification rate, unlike the length of the CNV: 75% of CNVs downgraded to benign or likely benign are less than 500 kb in size.

**Conclusions:**

This study’s high rate of reinterpretation suggests that CNV interpretation has rapidly evolved since 2010, thanks to the continuous enrichment of available databases. The reinterpreted CNV explained the phenotype for ten patients, leading to optimal genetic counseling. These findings suggest that CNVs should be reinterpreted at least every 2 years.

**Supplementary Information:**

The online version contains supplementary material available at 10.1186/s13073-023-01191-6.

## Background

Copy-number variations (CNVs) are a major cause of Mendelian disorders [1]. CNV detection using Array-CGH and SNP-array has revolutionized the diagnostic approach and identified the molecular basis of many genetic diseases [2, 3]. Therefore, Array-CGH has become an essential routine diagnostic tool for various indications, including developmental disabilities and congenital anomalies [4]. It has replaced conventional cytogenetic methods for most conditions [2] leading to a rapid increase in the detection of new microdeletion and microduplication syndromes [5]. More recently, the advent of next-generation sequencing (NGS) led to the detection of much smaller events (down to a single exon) thanks to the development of novel tools [6, 7].

An increase in the number of detected CNVs, especially short-sized, has highlighted the need for a comprehensive classification to assess the relationship between a CNV and a given phenotype. The American College of Medical Genetics (ACMG) and Clinical Genome Resource (ClinGen) have jointly proposed guidelines for standardizing the interpretation of copy number variants [8–10]. For example, a CNV is reported of uncertain significance when the available evidence is insufficient to determine clinical relevance unequivocally. Such CNVs can include, for example, a CNV described with conflicting interpretations in multiple publications or databases.

Small CNVs that include few genes are difficult to characterize because the number of overlapping CNVs described in the literature is statistically more limited. Consequently, they less frequently lead to a diagnosis. Thus, the increasing resolution of aCGH and SNP-array has led to the detection of more VUS. However, existing databases expand periodically, such as the database of genomic variants (DGV) [11] or Clinvar [12]. This new data adds potentially new information to interpret a given CNV, justifying a reanalysis.

VUS is a recurrent problem in medical genetics. First, a VUS does not lead to a conclusive diagnosis or appropriate genetic counseling. Management and treatment are thus difficult to define. Secondly, it is difficult for the patient to deal with uncertainty concerning VUS’s impact on clinical management [13, 14]. Finally, it is also challenging for the medical geneticist to decide whether to pursue diagnostic investigations.

As CNV classification largely depends on the available literature, local initiatives have led to the regular updating of CNV classification [15, 16]. Similar work has been done regarding NGS data and concluded the usefulness of systematic reanalysis with an interval greater than 18 months from the original report [17, 18]. However, no clear recommendations have been published regarding the optimal time to CNV reanalysis, and the effectiveness of systematic reinterpretation has not yet been assessed [19, 20]. Altogether, these observations highlight the need for guidelines for CNV reinterpretation. This study examined the usefulness of reinterpreting CNVs of uncertain significance.

## Methods

### Study design and characteristics of the cohorts

This retrospective monocentric French study covers from January 2010 to December 2017. The cohort was composed of all patients seen by a medical geneticist, for whom array-CGH testing was performed at CHRU Nancy’s medical genetics laboratory (1641 patients, Additional file 1: Fig. S1). Only patients who agreed to participate in the study are reported. The final cohort included 259 patients with CNVs of uncertain significance (Additional file 1: Fig S2, Additional file 1: Table S1).

### DNA samples

During routine care, peripheral blood, chorionic villi, or amniotic fluid were collected from the proband and peripheral blood from their parents (when available). DNA was extracted using the QIAmp DNA Kit (QIAGEN) manually or using the QIAcube instrument according to the manufacturer’s instructions.

### Array-based comparative genomic hybridization

aCGH was done using 180 K-oligonucleotide arrays (Agilent, San Clara, CA) with an average resolution of 25 kb. DNA preparation and hybridization procedures were performed according to the manufacturer’s instructions. Data were analyzed using genome build NCBI36/hg18 until 2011 or GRCh37/hg19 from 2012. If a chromosomal aberration was detected, further studies were performed using complementary methods (e.g., FISH, qPCR) depending on the finding [21] (Additional file 1: Fig. S1). Agilent CytoGenomics software was used to visualize CNVs. Finally, CNVs were interpreted using Cartagenia (Bench Lab CNV, Agilent) with DECIPHER and DGV databases and UCSC tools. An analysis threshold was set in 2016 in our lab to reduce VUS detection to improve diagnosis delay (500 kb in prenatal and 200 kb in postnatal).

### Data re-analyses

372 CNVs were interpreted independently by two genetic biologists specializing in neurodevelopmental diseases between November 2019 to March 2020. Results were then discussed with the clinical geneticists that prescribed the original analysis during a multidisciplinary meeting. No systematic approach of reanalysis was performed yet in our center explaining why re-classification was not done at patient follow-up.

NCBI36/hg18 CNVs boundaries were converted to GRCh37/hg19 build using the liftOver UCSC tool [22] (https://genome.ucsc.edu/cgi-bin/hgLiftOver). CNVs were then annotated by AnnotSV tool version 3.2.3 [23, 24]. In parallel, an MS Excel workbook listing all CNVs was built to allow efficient, standardized analysis accessible from any computer station. Several hyperlinks to public databases were created based on the genomic coordinate, variant type, and gene content. The following databases were interrogated: UCSC genome browser [22] (http://genome.UCSC.edu), the database of genomic variants[11] (http://dgv.tcag.ca/), OMIM (https://omim.org/), Clingen (https://clinicalgenome.org/)[25], Clinvar[12] (https://www.ncbi.nlm.nih.gov/clinvar/), filtering CNVs with at least one star (classification criteria provided) and DECIPHER database [26] (https://decipher.sanger.ac.uk/). Finally, a thorough Pubmed (https://pubmed.ncbi.nlm.nih.gov/) search was performed based on (1) genes included in the CNV and (2) the chromosome band and type of CNV (deletion/duplication).

**Fig. 1:**
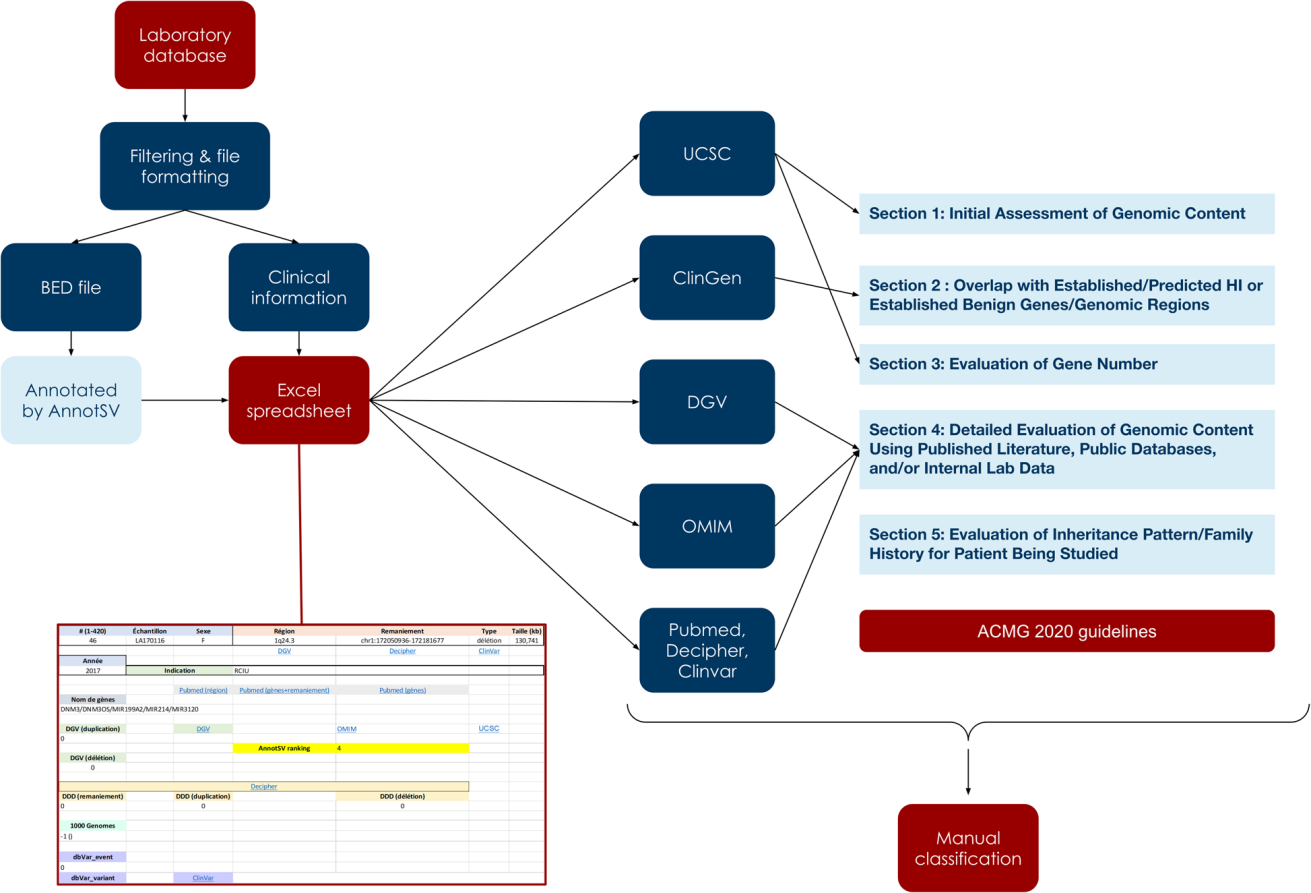
Representation of our approach during reanalysis. The workflow used is based on the 2020 ACMG recommendations (see sections on the right). After extraction of the list of aCGH analysis, we extracted CNVs classified as uncertain significance (left of the figure). This list allow us to create a bed file that was fed into AnnotSV website. Based on the resulting file and clinical information (gender, phenotype), we build an Excel spreadsheet summarizing both files and creating links to major online databases. Briefly, the gene content and frequency in the general population were checked using UCSC and DGV. ClinGen was used to determine dosage sensitivity and OMIM to assess possible morbid gene in the region. Finally, reported association was assessed using PubMed, Decipher, and Clinvar. All this data was combined in one slide presentation and analyzed using 2020 ACMG recommendations and ClinGen expert reports if available

Each CNV was then manually curated and classified as pathogenic, likely pathogenic, likely benign, benign, or of uncertain significance according to ACMG/ClinGen guidelines [8–10]. The workflow of our approach is summarized in Fig. 1.

### Statistical methods

Statistical analysis was performed using R software (R version 3.6.0, 2019-04-26 [27]). Percentages included only cases with known data for each feature — cases with missing data were excluded. Statistical tests included R2, linear regression, and comparison of the slopes (*P* < .05 was considered statistically significant).

### Ethical issues

This study was registered on clinicaltrials.gov (NCT04575350). Authorizations from patients, or their parents, were obtained at the time of genetic analysis with a signed consent form. As patients are systematically notified that their results may change over time, no specific consent for this study was signed. Ethical approval for this study was obtained from CHRU Nancy’s local ethical committee.

## Results

Of the 1641 array-CGH, 259 patients (15.8%) with 372 CNVs of uncertain significance were documented (Additional file 1: Fig. S2, Additional file 1: Table S1). Tests ranged from the prenatal period to a 72-year-old patient. Male and female patients were equally represented. Most patients were seen for malformation, intellectual disability, or autism (Additional file 1: Table S1).

**Fig. 2:**
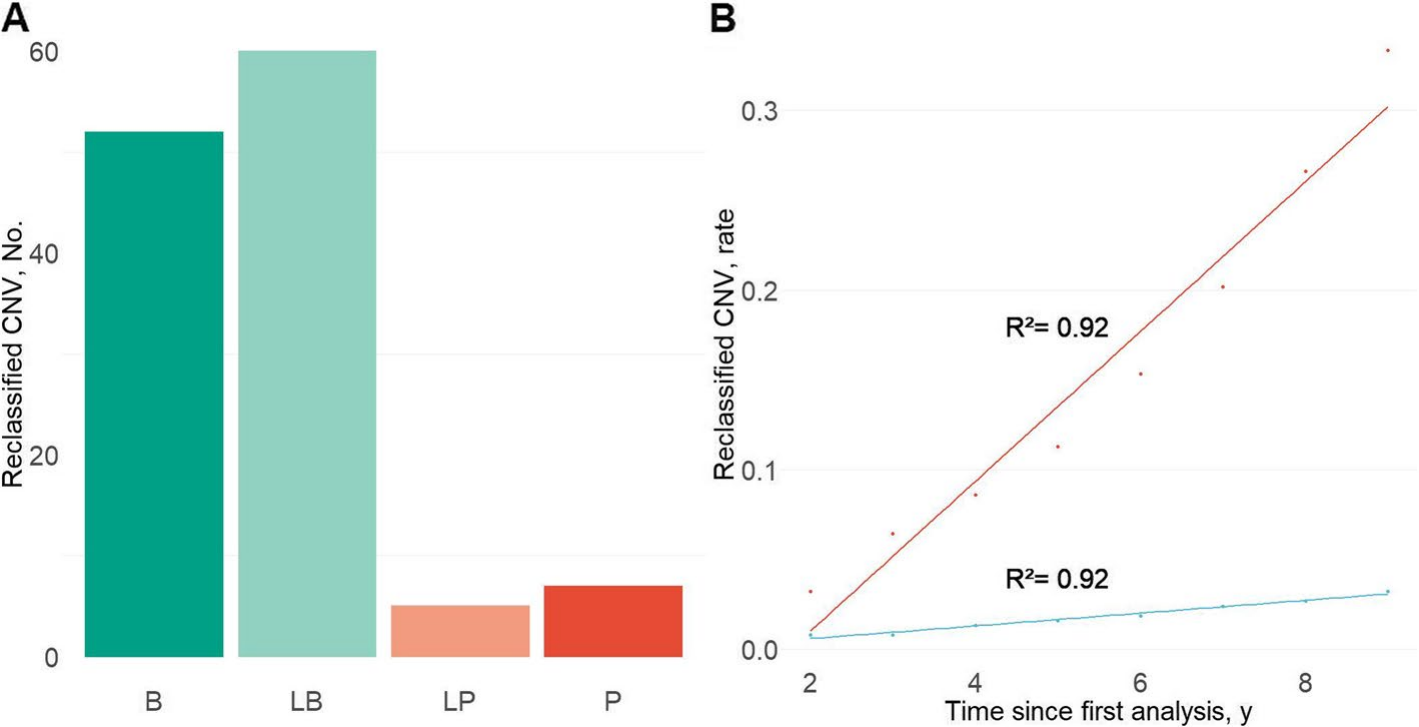
VUSs With Variant Classification changes. **A** Patients with reclassification of CNVs from each of the categories. 52 CNVs of uncertain significance were reclassified as benign, 60 as likely benign, 5 as likely pathogenic, and 7 as pathogenic. **B** Cumulative reclassification rate plotted as the cumulative fraction of reclassified variants for each year. Testing was calculated using either only pathogenic and likely pathogenic CNVs (blue line) or all reclassified CNVs (red line). The extrapolated slopes for the change in VUS classification are 4.2% per year and 0.4% respectively. This linear distribution means that reanalysis rate is constant every year. The R^2^ and slope values were calculated using linear regression

After re-analyzing all cases with reported VUS, 106 of the 259 patients (40.9%) had a revised classification. 112 CNVs were downgraded in pathogenicity (30.1%, 52 to benign, and 60 to likely benign), and 12 were upgraded (3.2%, 5 likely pathogenic, and 7 pathogenic, Fig. 2, Table 1). 76% of CNVs first reported as VUS were smaller than 500 kb (Additional file 1: Table S2). Notably, in later years, CNVs of bigger sizes make up a larger proportion of the findings (1/46, 2% CNVs > 500 kb in 2010 and 18/48, 38% in 2017). Deletions and duplications were found in equal proportions (54% vs. 46%, respectively). Most CNVs (86.5% of CNVs when the information was known) were inherited.

**Table 1:** Patients with a CNV previously described as VUS classified as pathogenic and likely pathogenic

**CNV arr [hg19]**	**Type**	**Transmission**	**New class**	**ACMG score**	**CNV nb.**	**Related to the primary medical reason for testing**	**Indication**	**Gender**	**Age (year)**
*CNVs with full penetrance*									
1q24.3(172050936-172181677)x1	Deletion	Maternal	LP	0.9	1	Yes	Intrauterine growth restriction	F	-
Xq26.2(132717085-132924462)x1	Deletion	*De novo*	P	1	4	Yes	Delay of acquisitions	F	19
*CNVs with incomplete penetrance*									
1q21.1(145818702-147824207)x3	Duplication	*De novo*	P	1	1	Partially	Vaginal aplasia with unilateral renal agenesis and benign myoclonic epilepsy	F	2
4q31.23(148911418-149103259)x1	Deletion	Maternal	LP	0.9	1	Partially	Intellectual disability with pseudohypoaldosteronism	F	19
Xq27.1(139103383-139763381)x2~3	Duplication-triplication	Maternal	LP	0.9	2	Yes	Acrania	F	-
Xq27.1(139103383-139801281)x2	Duplication	Maternal	LP	0.9	1	Yes	Spina bifida	F	-
*Neurodevelopmental predisposing factors*									
2p16.3(51172123-51314430)x1	Deletion	Paternal	P	1	1	Yes	Speech and language delay in a family context	F	10
2p16.3(51251498-51491417)x1	Deletion	Paternal	P	1	1	Yes	Delayed acquisitions, behavioral disorders	M	7
2q12.3q13(109320835-110427254)x1	Deletion	Non-maternal	LP	0.9	2	Yes	Delay of acquisitions and autism traits	M	13
15q11.2(22765628-23191062)x1	Deletion	Maternal	P	1	1	Yes	Delay of acquisitions	F	6
16p11.2(28615644-29042118)x3	Duplication	Paternal	P	1	2	No	Malformations	M	0
17q12(34817422-36243028)x3	Duplication	Paternal	P	1	1	No	Ice pick feet	F	-

“Non-maternal” means that the CNV was not inherited by the mother and that father was not available to be tested. The column “CNV nb.” indicates the number of CNV detected in the studied patient

Next, we examined the likelihood of reclassification over time. CNVs were reclassified as benign or likely benign because of new DGV information. In contrast, CNVs were reclassified as pathogenic or likely pathogenic thanks to ClinGen or new literature referenced in Pubmed. All CNV reclassified as pathogenic or likely pathogenic were classified as such by AnnotSV. The reclassification rate varies greatly when considering all CNVs reclassified or only CNV reclassified as pathogenic or likely pathogenic (P-LP, 0.4 % change per year; all VUS, 4.2% change per year, *p*=0.003, Fig. 2).

**Fig. 3:**
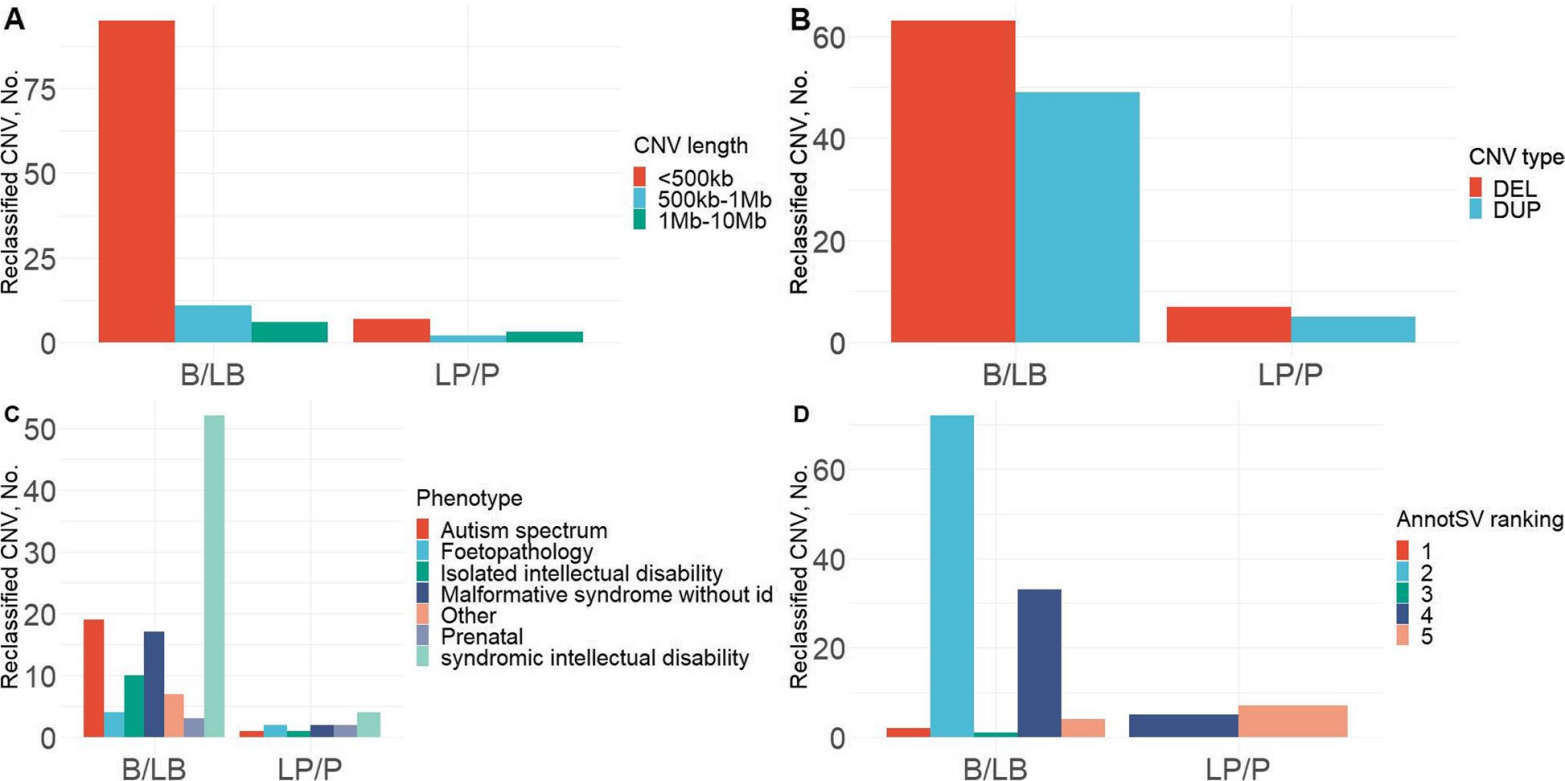
Variants reinterpreted in the study. Number of reclassified CNVs depending on CNV size (**A**), CNV type (**B**), patient phenotype (**D**), or CNV AnnotSV ranking regarding downgraded (B/LB) or upgraded (LP/P) CNVs

Most CNVs reclassified as benign were short (<500kb, *n*=95, Fig. 3). However, no association existed between CNV type and class change.

The twelve CNVs reclassified as pathogenic or likely pathogenic are summarized in Table 1. Three groups are distinguished: (1) CNVs with full penetrance; (2) CNVs with incomplete penetrance; (3) neurodevelopmental predisposing factors.

A *de novo* Xq26.2 deletion was identified in 2010 in a 19-year-old girl with mild intellectual disability who was also heterozygous for the familial 21q21.1 duplication already known to be associated with her deficiency. Random X inactivation was noted (78%/22%). This Xq26.2 deletion includes the minimal critical region of Simpson-Golabi-Behmel syndrome, especially *GPC3 *[28–31]. Milder forms of this X-linked syndrome have been previously reported in female patients [30]; only four female cases are now documented in the literature. Increased incidence of neoplasia described in this syndrome would need to be monitored [32].

In 2017, we identified a 1q24.3 deletion in a fetus presenting with Intra Uterine Growth Restriction (IUGR). This CNV was inherited from his mother. During our reanalysis, we saw that this CNV was newly reported with short stature, microcephaly, brachydactyly, dysmorphic facial features, and intellectual disability [33–35]. Two inherited cases have been previously reported [34]. Our deletion containing part of the *DNM3* gene, miR199, and miR214 that are harbored within intron 14, was included in the minimal region linked in 2018 to the syndrome [34]. Moreover, the pregnant mother also presented this skeletal phenotype, as she measured 1.54 meters and had brachydactyly.

Four CNVs are described with incomplete penetrance.

A small deletion of 191 kb in 4q31.23, detected in 2010, was reclassified following our work as likely pathogenic in a girl presenting with global developmental delay and pseudohypoaldosteronism. She inherited the deletion from her mother. This deletion contains *NR3C2* (OMIM* 600983), encoding an aldosterone receptor. The implication of this CNV in pseudohypoaldosteronism was suspected, and conclusive evidence is now available [36–40]. The mother did not present symptoms, and asymptomatic patients have already been reported [39]. However, this CNV does not explain the neurodevelopmental delay.

Two copy gain CNVs encompassing *SOX3* were found in 2015 and 2017 in our cohort in two fetuses: one duplication in a male fetus presenting with spina bifida (arr [hg19] Xq27.1(139103383_139801281)x2) and a duplication-triplication in a male fetus with acrania (arr [hg19] Xq27.1(139103383-139763381)x2~3). *SOX3* duplications are implicated in variable phenotypes, including myelomeningocele in both sexes, intellectual disability (of varying severity), and growth hormone deficiency (including panhypopituitarism) in males [41]. Hureaux et al., 2019 conducted a study on a fetal cohort showing that these *SOX3* gene duplications are involved in neural tube closure defects [42]. To our knowledge, *SOX3* duplication-triplication has never been reported.

One of the CNVs, a *de novo* 1q21.1 duplication (arr [hg19] chr1:(145818702-147824207)x3 dn, SCV001480529), was identified in a 2-year-old girl presenting with vaginal aplasia, unilateral renal agenesis, and benign myoclonic epilepsy in 2011. This duplication is larger than the classical 1q21.1 duplication syndrome (distal, hg19, chr1:146577486-147394506). The extension of the recurrent duplication is of particular interest as microdeletions, and microduplications of the distal 1q21.1 region have been linked, after the initial analysis, to various disorders, including Mayer–Rokitansky–Küster–Hauser syndrome (MRKH MIM% 277000), and autism [43, 44]. MRKH is a congenital malformation characterized by impaired Müllerian duct development resulting in a missing uterus and variable degrees of upper vaginal hypoplasia. Chen and colleagues reported a woman presenting MRKH associated with a 31.48 kb (chr1:146778208-146809687) deletion [34]. As no duplications have been reported in this region, we could still not conclude that this CNV explained our patient’s malformations. However, we considered this CNV pathogenic, at least a recurrent neurodevelopmental predisposing factor, thanks to proven haploinsuffisance sensitivity. Myoclonic epilepsy could be linked to the 1q21.1 duplication syndrome.

Six of the twelve likely pathogenic or pathogenic CNVs are neurodevelopmental disorder/autism spectrum disorder (ASD) predisposing factors, four of which explain part of the patient’s pathology (Table 1). Three were recurrent variations, reclassified thanks to detailed descriptions on ClinGen’s “Recurrent CNV” list (https://search.clinicalgenome.org/kb/gene-dosage/cnv: one CNV is a 15q11.2 deletion, one a 16p11.2 duplication, and one a 17q12 duplication. Two other patients harbored a 2p16.2 deletion, diagnosed in 2012, including *NRXN1* curated with sufficient evidence of haploinsufficiency and strong proof of pathogenicity in 2017 [45, 46]. One patient had a heterozygous 1,106 Mb deletion on 2q12.3q13 identified in 2014. It was recently associated with developmental delay and behavioral problems [47].

## Discussion

During this 8-year study period,106 of the 259 patients (40.9%) had revised CNV classification. In our center, VUS had a yearly reclassification rate of 4.2%.

A clinically significant change occurred for 12 patients (4.6%).

Ten CNVs reclassified as pathogenic or likely pathogenic explained, at least partially, the patients' pathology (Table 1). These diagnoses have two main consequences: (1) clarification of the pathogenicity of the CNV allows appropriate genetic counseling (2) molecular diagnosis alleviates the need for lengthy and often (very) expensive analyses, and decreasing invasive and painful procedures helps compliance and follow-up which is an additional benefit. Moreover, from an economic point of view, reanalysis is far more cost-effective than doing a new analysis. These statements need to be mitigated for some patients and, in particular, those harboring predisposing factors (half of the patients in our study). For these patients, additional tests are needed such as exome sequencing.

Finally, in our study, reclassifications to pathogenic and likely pathogenic were due to the publication of new papers, highlighting the need for teams to publish cases and for collaborative works. International cooperations and studies are crucial aspects of VUS reinterpretation.

Among the twelve CNVs reclassified as pathogenic or likely pathogenic, six are predisposing factors for neurodevelopmental disorder/autism spectrum disorder (ASD). The complexity of interpreting these CNVs is related to the inherent difficulty in assessing their level of involvement in the patient’s phenotype and whether further genetic testing is warranted. The reason for reclassification was the publication of reports by expert groups confirming the role of predisposing factors (15q11 region, dosage ID: ISCA-37448, for example). Our work highlights the importance of the ClinGen dosage sensitivity map that has already been used for ClinVar CNV reclassification [48].

For 112 (30.2%) CNVs, a link with the patient’s phenotype was ruled out. The revised interpretation was due to an overlapping CNV in the general population at least in one cohort. Most of these CNVs had no gene content or were small intragenic CNVs away from the coding sequence. Significant changes were linked to DGV enrichment over the years (http://dgv.tcag.ca/dgv/app/downloads?ref=GRCh37/hg19#articles_cited). As has already been frequently stated, the identification of VUS has a significant impact on both patient and practitioner: stressful announcement, uncertainty as to what action to take, incomplete genetic counseling, or even difficulty in knowing what level of information to divulge [49, 50]. In our study, we could inform half of the patients concerning the non-pathogenicity of their variation.

Some CNVs were of interest. We report a new female case with Simpson-Golabi-Behmel syndrome. We highlight the possible association between a specific portion of 1q21.1 deletion and MRKH syndrome. Moreover, we further delineate the 1q24.3 deletion and highlight the role of two microRNAs (miR199 and miR214) located within intron 14 of *DNM3*. The role of *SOX3* in neural tube defects is also reinforced by two copy number gains associated with acrania and spina bifida.

In this work, we propose an efficient strategy for CNV reanalysis with reproducibility in the analysis method and the tools used.

As all the CNVs reclassified as (likely) pathogenic were identified as such by AnnotSV, the sensitivity of this tool has been demonstrated (Additional file 1: Table S3, specificity = 75%, sensitivity = 100%). Indeed, on the 372 CNVs for which a reanalysis is required, only 102 (20 + 82) should be manually curated. As such, including AnnotSV in the workflow reduces the number of CNVs to be manually analyzed by almost four if considering only CNVs classified by AnnotSV as pathogenic or likely pathogenic. However, the precision remains low: on the 102 positive cases, only 12 (11.8%) were correctly classified as (likely) pathogenic.

As the rate of reinterpretation seems constant over the years, we cannot determine a specific delay for efficient reanalysis (Fig. 2). Based on previous reports analyzing NGS data re-interpretation [51], we started our work two years after the latest report. Consequently, we recommend reinterpretation of CNV at a minimum frequency of every 2 years. Implementing an automatic monitoring system would be also a solution [52]. CNV reclassified as pathogenic or likely pathogenic did not have specific characteristics. Consequently, the reinterpretation should not be limited to *de novo CNVs*, large CNVs, or copy number loss.

The monocentric design limited this study. Moreover, our results are mitigated by the high prevalence of CNV with risk factors. Further investigations are needed for these patients. In addition, it is unclear whether such a high rate of VUS reclassified as benign will remain stable as the DGV database is now more comprehensive than in 2010.

## Conclusions

In conclusion, based on our long-termed experience of CGH array analysis, systematic reanalysis of CNVs of uncertain significance should be considered standard practice for all genetics laboratories. In summary, patients with CNV of uncertain significance should have their results reinterpreted at least every two years and before further genetic testing. The clinician should warn the patient at the time of the prescription that the outcome may change depending on the state of knowledge. Moreover, as no fully automatic system is yet available and based on ACMG guidelines, it should also be the responsibility of the clinician to prescribe such reanalysis at each follow-up consultation. Of course, points raised in this article on the reinterpretation of array CGH also apply to the reinterpretation of CNV detected by NGS analyses.

## Supplementary Information

**Additional file 1:** **FigureS1.** Array-CGH interpretation workflow. **Figure S2.** Flowchart indicating all samples included in our study. aCGH: array comparative genomic hybridization; CNV: copy number variation; VUS:variant of uncertain significance. AnnotSV was applied to the whole VUS cohort. We then compare the automatic ACMG classification from AnnotSV to our own manual classification. Missing data correspond to patient for whome definitive CNV classification was not stated on the first biologist report or that the conclusion was not reported on our database. **Table S1. **Characteristics of the cohort composed of the 259 patients with a VUS identified on array-CGH. Only 180k array-CGH platform were used for this study. **Table S2.** Characteristics of CNV first reported as VUS. B: benign, LB: likely benign; VUS: variantof uncertain significance; LP: likely pathogenic; P: pathogenic. **Table S3.** AnnotSV performance. Contingency table of classification proposed by AnnotSV versus our classification for the372 CNVs primarily reported as VUS.

## Data Availability

The variant information generated and analyzed in this study are accessible at the ClinVar (https://www.ncbi.nlm.nih.gov/clinvar/) and DECIPHER websites (https://www.deciphergenomics.org/) under the following accession numbers: SCV001480526 - SCV001480573 and SCV001547200 - SCV001547241 and DECIPHER 424280-424328 and 428935-428976.

## References

[1] Itsara A, Wu H, Smith JD (2010). De novo rates and selection of large copy number variation. Genome Res.

[2] Miller DT, Adam MP, Aradhya S (2010). Consensus statement: chromosomal microarray is a first-tier clinical diagnostic test for individuals with developmental disabilities or congenital anomalies. Am J Hum Genet.

[3] Manning M, Hudgins L (2010). Array-based technology and recommendations for utilization in medical genetics practice for detection of chromosomal abnormalities. Genet Med.

[4] Hureaux M, Guterman S, Hervé B (2019). Chromosomal microarray analysis in fetuses with an isolated congenital heart defect: a retrospective, nationwide, multicenter study in France. Prenat Diagn.

[5] Shaffer LG, Bejjani BA, Torchia B, Kirkpatrick S, Coppinger J, Ballif BC (2007). The identification of microdeletion syndromes and other chromosome abnormalities: cytogenetic methods of the past, new technologies for the future. Am J Med Genet Part C Semin Med Genet..

[6] Tan R, Wang Y, Kleinstein SE (2014). An evaluation of copy number variation detection tools from whole-exome sequencing data. Hum Mutat.

[7] Roca I, González-Castro L, Fernández H, Couce ML, Fernández-Marmiesse A (2019). Free-access copy-number variant detection tools for targeted next-generation sequencing data. Mutat Res.

[8] Kearney HM, Thorland EC, Brown KK, Quintero-Rivera F, South ST (2011). American College of Medical Genetics standards and guidelines for interpretation and reporting of postnatal constitutional copy number variants. Genet Med.

[9] Riggs ER, Andersen EF, Cherry AM (2020). Technical standards for the interpretation and reporting of constitutional copy-number variants: a joint consensus recommendation of the American College of Medical Genetics and Genomics (ACMG) and the Clinical Genome Resource (ClinGen). Genet Med.

[10] Brandt T, Sack LM, Arjona D (2020). Adapting ACMG/AMP sequence variant classification guidelines for single-gene copy number variants. Genet Med.

[11] MacDonald JR, Ziman R, Yuen RK, Feuk L, Scherer SW (2014). The Database of Genomic Variants: a curated collection of structural variation in the human genome. Nucleic Acids Res.

[12] Landrum MJ, Lee JM, Benson M (2018). ClinVar: improving access to variant interpretations and supporting evidence. Nucleic Acids Res.

[13] Makhnoon S, Garrett LT, Burke W, Bowen DJ, Shirts BH (2019). Experiences of patients seeking to participate in variant of uncertain significance reclassification research. J Commun Genet.

[14] Jez S, Martin M, South S, Vanzo R, Rothwell E (2015). Variants of unknown significance on chromosomal microarray analysis: parental perspectives. J Community Genet.

[15] van Luttikhuizen JL, Bublitz J, Schubert S (2020). From a variant of unknown significance to pathogenic: Reclassification of a large novel duplication in BRCA2 by high-throughput sequencing. Mol Genet Genomic Med.

[16] Lattimore V, Currie M, Lintott C, Sullivan J, Robinson BA, Walker LC (2015). Meeting the challenges of interpreting variants of unknown clinical significance in BRCA testing. N Z Med J.

[17] Ewans LJ, Schofield D, Shrestha R (2018). Whole-exome sequencing reanalysis at 12 months boosts diagnosis and is cost-effective when applied early in Mendelian disorders. Genet Med.

[18] Bruel AL, Nambot S, Quéré V (2019). Increased diagnostic and new genes identification outcome using research reanalysis of singleton exome sequencing. Eur J Hum Genet.

[19] Farooqi MS, Figueroa S, Gotway G, Wang J, Luu HS, Park JY (2020). Reinterpretation of Chromosomal Microarrays with Detailed Medical History. J Pediatr.

[20] Dai P, Honda A, Ewans L (2022). Recommendations for next generation sequencing data reanalysis of unsolved cases with suspected Mendelian disorders: a systematic review and meta-analysis. Genet Med.

[21] Livak KJ, Schmittgen TD (2001). Analysis of relative gene expression data using real-time quantitative PCR and the 2(-Delta Delta C(T)) Method. Methods (San Diego, Calif).

[22] Navarro Gonzalez J, Zweig AS, Speir ML (2021). The UCSC Genome Browser database: 2021 update. Nucleic Acids Res.

[23] Geoffroy V, Herenger Y, Kress A (2018). AnnotSV: an integrated tool for structural variations annotation. Bioinformatics (Oxford, England).

[24] Geoffroy V, Guignard T, Kress A (2021). AnnotSV and knotAnnotSV: a web server for human structural variations annotations, ranking and analysis. Nucleic Acids Res.

[25] Preston CG, Wright MW, Madhavrao R (2022). ClinGen Variant Curation Interface: a variant classification platform for the application of evidence criteria from ACMG/AMP guidelines. Genome Med.

[26] Chatzimichali EA, Brent S, Hutton B (2015). Facilitating collaboration in rare genetic disorders through effective matchmaking in DECIPHER. Hum Mutat.

[27] Team RC. R: A language and environment for statistical computing. Vienna: R Foundation for Statistical Computing; 2022.

[28] Andrysiak-Mamos E, Sagan KP, Lietz-Kijak D (2019). Simpson-Golabi-Behmel syndrome in a 39-year-old male patient with suspected acromegaly-A case study. Am J Med Genet Part A.

[29] Schmidt J, Hollstein R, Kaiser FJ, Gillessen-Kaesbach G (2017). Molecular analysis of a novel intragenic deletion in GPC3 in three cousins with Simpson-Golabi-Behmel syndrome. Am J Med Genet Part A.

[30] Vaisfeld A, Pomponi MG, Pietrobono R, Tabolacci E, Neri G (2017). Simpson-Golabi-Behmel syndrome in a female: A case report and an unsolved issue. Am J Med Genet Part A.

[31] Vuillaume ML, Moizard MP, Rossignol S (2018). Mutation update for the GPC3 gene involved in Simpson-Golabi-Behmel syndrome and review of the literature. Hum Mutat.

[32] Neri G, Gurrieri F, Zanni G, Lin A (1998). Clinical and molecular aspects of the Simpson-Golabi-Behmel syndrome. Am J Med Genet.

[33] Lefroy H, Fox O, Javaid MK, Makaya T, Shears DJ (2018). 1q24 deletion syndrome. Two cases and new insights into genotype-phenotype correlations. Am J Med Genet Part A..

[34] Chatron N, Haddad V, Andrieux J (2015). Refinement of genotype-phenotype correlation in 18 patients carrying a 1q24q25 deletion. Am J Med Genet Part A..

[35] Ashraf T, Collinson MN, Fairhurst J, Wang R, Wilson LC, Foulds N (2015). Two further patients with the 1q24 deletion syndrome expand the phenotype: A possible role for the miR199-214 cluster in the skeletal features of the condition. Am J Med Genet Part A..

[36] O'Connell SM, Johnson SR, Lewis BD (2011). Structural chromosome disruption of the NR3C2 gene causing pseudohypoaldosteronism type 1 presenting in infancy. J Pediatr Endocrinol Metab.

[37] Casas-Alba D, Vila Cots J, Monfort Carretero L (2017). Pseudohypoaldosteronism types I and II: little more than a name in common. J Pediatr Endocrinol Metab.

[38] Gopal-Kothandapani JS, Doshi AB, Smith K (2019). Phenotypic diversity and correlation with the genotypes of pseudohypoaldosteronism type 1. J Pediatr Endocrinol Metab.

[39] Hanukoglu A, Vargas-Poussou R, Landau Z, Yosovich K, Hureaux M, Zennaro MC (2020). Renin-aldosterone system evaluation over four decades in an extended family with autosomal dominant pseudohypoaldosteronism due to a deletion in the NR3C2 gene. J Steroid Biochem Mol Biol.

[40] Barone Pritchard A, Ritter A, Kearney HM, Izumi K (2020). Interstitial 4q Deletion Syndrome Including NR3C2 Causing Pseudohypoaldosteronism. Mol Syndromol.

[41] Butler KM, Fee T, DuPont BR, Dean JH, Stevenson RE, Lyons MJ (2022). A SOX3 duplication and lumbosacral spina bifida in three generations. Am J Med Genet Part A.

[42] Hureaux M, Ben Miled S, Chatron N (2019). SOX3 duplication: A genetic cause to investigate in fetuses with neural tube defects. Prenat Diagn.

[43] Rosenfeld JA, Traylor RN, Schaefer GB (2012). Proximal microdeletions and microduplications of 1q21.1 contribute to variable abnormal phenotypes. Eur J Hum Genet..

[44] Chen MJ, Wei SY, Yang WS (2015). Concurrent exome-targeted next-generation sequencing and single nucleotide polymorphism array to identify the causative genetic aberrations of isolated Mayer-Rokitansky-Küster-Hauser syndrome. Hum Reprod (Oxford, England).

[45] Lowther C, Speevak M, Armour CM (2017). Molecular characterization of NRXN1 deletions from 19,263 clinical microarray cases identifies exons important for neurodevelopmental disease expression. Genet Med.

[46] Al Shehhi M, Forman EB, Fitzgerald JE (2019). NRXN1 deletion syndrome; phenotypic and penetrance data from 34 families. Eur J Med Genet.

[47] Huynh MT, Gérard M, Ranguin K (2021). Novel interstitial 2q12.3q13 microdeletion predisposes to developmental delay and behavioral problems. Neurogenetics.

[48] Riggs ER, Nelson T, Merz A (2018). Copy number variant discrepancy resolution using the ClinGen dosage sensitivity map results in updated clinical interpretations in ClinVar. Hum Mutat.

[49] Hall MJ, Forman AD, Pilarski R, Wiesner G, Giri VN (2014). Gene panel testing for inherited cancer risk. J Natl Comprehensive Cancer Netw.

[50] Medendorp NM, Hillen MA, Murugesu L, Aalfs CM, Stiggelbout AM, Smets EMA (2019). Uncertainty related to multigene panel testing for cancer: a qualitative study on counsellors' and counselees' views. J Commun Genet.

[51] SoRelle JA, Thodeson DM, Arnold S, Gotway G, Park JY (2019). Clinical utility of reinterpreting previously reported genomic epilepsy test results for pediatric patients. JAMA Pediatr.

[52] Yauy K, Lecoquierre F, Baert-Desurmont S (2022). Genome Alert!: A standardized procedure for genomic variant reinterpretation and automated gene-phenotype reassessment in clinical routine. Genet Med.

