# Supplementary material for: Clinical utility of periodic reinterpretation of CNVs of uncertain significance: an 8-year retrospective study
Source: Genome Med. 2023 May 23;15:39. doi: 10.1186/s13073-023-01191-6 (PMC10204260; doi:10.1186/s13073-023-01191-6)
Supplement: Supplementary file 1 — Additional file 1: FigureS1. Array-CGH interpretation workflow. Figure S2. Flowchart indicating all samples included in our study. aCGH: array comparative genomic hybridization; CNV: copy number variation; VUS:variant of uncertain significance. AnnotSV was applied to the whole VUS cohort. We then compare the automatic ACMG classification from AnnotSV to our own manual classification. Missing data correspond to patient for whome definitive CNV classification was not stated on the first biologist report or that the conclusion was not reported on our database. Table S1. Characteristics of the cohort composed of the 259 patients with a VUS identified on array-CGH. Only 180k array-CGH platform were used for this study. Table S2. Characteristics of CNV first reported as VUS. B: benign, LB: likely benign; VUS: variantof uncertain significance; LP: likely pathogenic; P: pathogenic. Table S3. AnnotSV performance. Contingency table of classification proposed by AnnotSV versus our classification for the372 CNVs primarily reported as VUS. [file 13073_2023_1191_MOESM1_ESM.pdf]

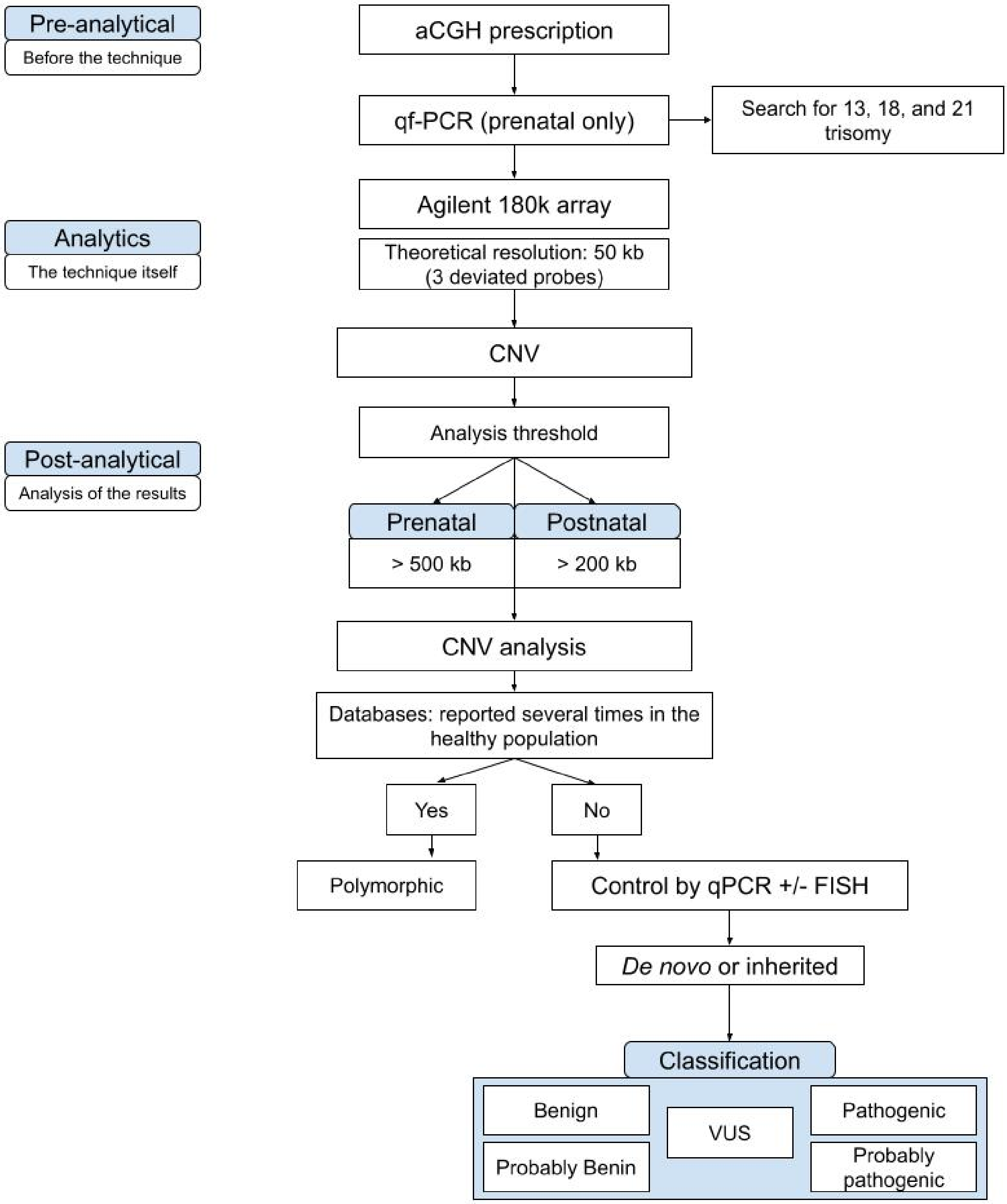


**Figure S1. Array-CGH interpretation workflow.**

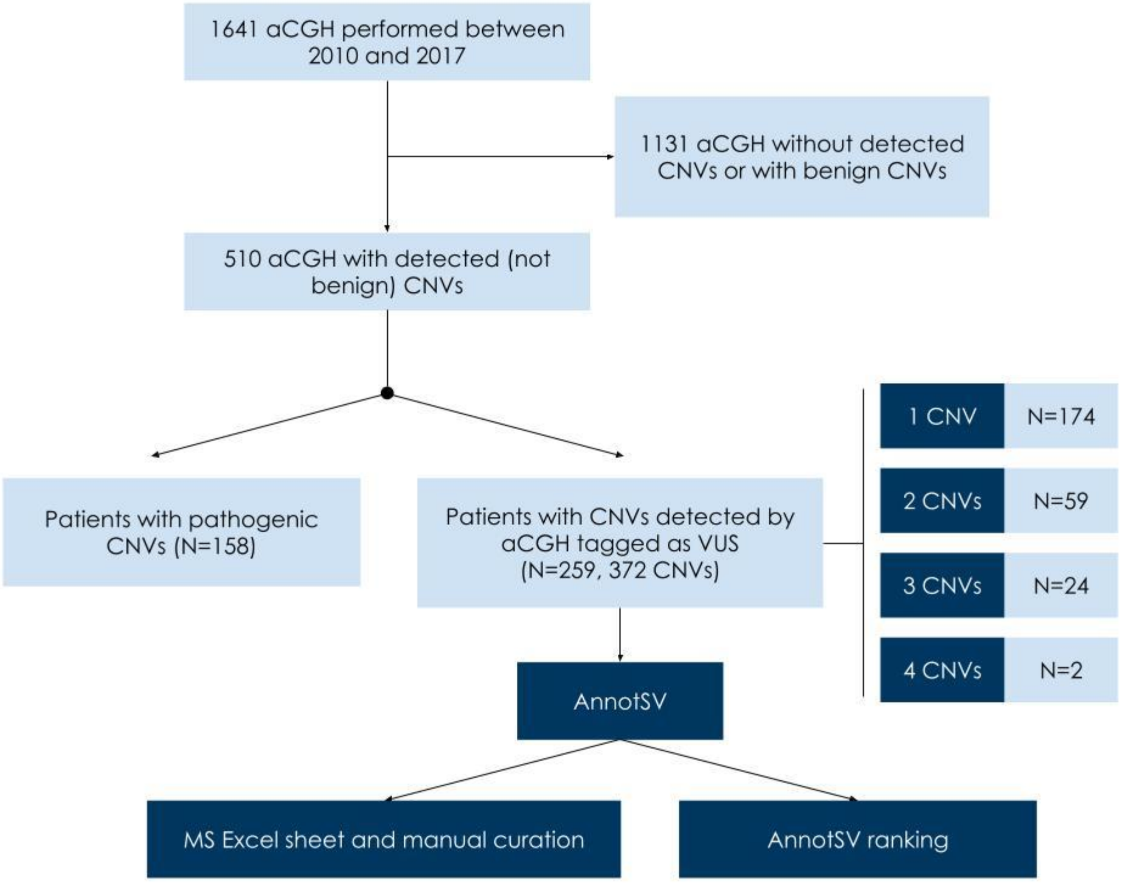


**Figure S2. Flowchart indicating all samples included in our study**. aCGH: array comparative genomic hybridization; CNV: copy number variation; VUS: variant of uncertain significance. AnnotSV was applied to the whole VUS cohort. We then compare the automatic ACMG classification from AnnotSV to our own manual classification (also based on the 2020 recommandation). Missing data correspond to patient for whome definitive CNV classification was not stated on the first biologist report or that the conclusion was not reported on our database.

**Table S1. Characteristics of the cohort composed of the 259 patients with a VUS identified on array CGH**. Only 180k array CGH platform were used for this study.

| | Total (N = 259) |
|---|---|
| **Sex** | |
| F | 127 (49%) |
| M | 132 (51%) |
| **Age** | |
| minimum | 0 |
| median (IQR) | 7.10 (3.30, 19.20) |
| mean (sd) | 11.91 ± 11.97 |
| maximum | 72.7 |
| **Indication** | |
| Syndromic intellectual disability | 79 (31%) |
| Autism spectrum | 48 (19%) |
| Malformative syndrome without intellectual disability | 39 (15%) |
| Isolated intellectual disability | 37 (14%) |
| Prenatal | 26 (10%) |
| Foetopathology | 10 (4%) |
| Unknown/Missing | 3 (1.16%) |
| Chromosomic abnormality | 1 (0%) |
| Other | 16 (6%) |

**Table S2. Characteristics of CNV first reported as VUS.** B: benign, LB: likely benign; VUS: variant of uncertain significance; LP: likely pathogenic; P: pathogenic

| | Total (N = 372) | 2010 (N = 47) | 2011 (N = 49) | 2012 (N = 39) | 2013 (N = 46) | 2014 (N = 39) | 2015 (N = 37) | 2016 (N = 67) | 2017 (N = 48) |
|---|---|---|---|---|---|---|---|---|---|
| **Ranking** | | | | | | | | | |
| B | 52 (14) | 8 (17) | 15 (31) | 10 (26) | 5 (11) | 4 (10) | 0 (0) | 7 (10) | 3 (6) |
| LB | 60 (16) | 15 (32) | 8 (16) | 6 (15) | 9 (20) | 5 (13) | 6 (16) | 5 (7) | 6 (12) |
| VUS | 248 (67) | 22 (47) | 25 (51) | 21 (54) | 31 (67) | 29 (74) | 29 (78) | 55 (82) | 36 (75) |
| LP | 5 (1) | 1 (2) | 0 (0) | 0 (0) | 0 (0) | 1 (3) | 1 (3) | 0 (0) | 2 (4) |
| P | 7 (2) | 1 (2) | 1 (2) | 2 (5) | 1 (2) | 0 (0) | 1 (3) | 0 (0) | 1 (2) |
| **Type** | | | | | | | | | |
| Deletion | 200 (54) | 23 (49) | 29 (59) | 29 (74) | 27 (59) | 21 (54) | 15 (41) | 32 (48) | 24 (50) |
| Duplication | 172 (46) | 24 (51) | 20 (41) | 10 (26) | 19 (41) | 18 (46) | 22 (59) | 35 (52) | 24 (50) |
| **Transmission** | | | | | | | | | |
| *de novo* | 31 (13) | 6 (19) | 3 (12) | 2 (10) | 4 (12) | 3 (10) | 1 (6) | 8 (20) | 4 (13) |
| maternal | 125 (54) | 16 (52) | 16 (62) | 6 (30) | 21 (64) | 13 (42) | 11 (61) | 23 (58) | 19 (61) |
| paternal | 74 (32) | 9 (29) | 7 (27) | 12 (60) | 8 (24) | 15 (48) | 6 (33) | 9 (22) | 8 (26) |
| Unknown/Missing | 142 (38.17%) | 16 (34.04%) | 23 (46.94%) | 19 (48.72%) | 13 (28.26%) | 8 (20.51%) | 19 (51.35%) | 27 (40.30%) | 17 (35.42%) |
| **Length** | | | | | | | | | |
| <500kb | 281 (76) | 46 (98) | 40 (82) | 32 (82) | 39 (85) | 27 (69) | 20 (54) | 47 (70) | 30 (62) |
| >500kb <1Mb | 40 (11) | 1 (2) | 5 (10) | 5 (13) | 4 (9) | 2 (5) | 7 (19) | 7 (10) | 9 (19) |
| >1Mb <10Mb | 51 (14) | 0 (0) | 4 (8) | 2 (5) | 3 (7) | 10 (26) | 10 (27) | 13 (19) | 9 (19) |
| >10Mb | 0 (0) | 0 (0) | 0 (0) | 0 (0) | 0 (0) | 0 (0) | 0 (0) | 0 (0) | 0 (0) |

**Table S3. AnnotSV performance.** Contingency table of classification proposed by AnnotSV versus our classification for the 372 CNVs primarily reported as VUS

| | | Manual ranking | | | | | |
|---|---|---|---|---|---|---|---|
| | | **1** | **2** | **3** | **4** | **5** | **Sum** |
| AnnotSV | **1** | 4 | | 1 | | | 5 |
| | **3** | 42 | 53 | 170 | | | 265 |
| | **4** | 1 | 2 | 17 | | | 20 |
| | **5** | 5 | 5 | 60 | 5 | 7 | 82 |
| | **Sum** | 52 | 60 | 248 | 5 | 7 | 372 |
